# Histone Modification of Embryonic Stem Cells Produced by Somatic Cell Nuclear Transfer and Fertilized Blastocysts

**Published:** 2013-11-20

**Authors:** Fattaneh Farifteh, Mohammad Salehi, Mojgan Bandehpour, Mosaffa Nariman, Marefat Ghafari Novin, Taher Hosseini, Sedigheh Nematollahi, Mohsen Noroozian, Somayeh Keshavarzi, Ahmad Hosseini

**Affiliations:** 1 Cellular and Molecular Biology Research Center, Faculty of Medicine, Shahid Beheshti University of Medical Science, Tehran, Iran; 2 Department of Cell Biology and Anatomical Science, Faculty of Medicine, Shahid Beheshti University of Medical Sciences, Tehran, Iran; 3 Department of Embryology, Mehr Infertility Institute, Rasht, Iran; 4 Department of Biothechnology, Faculty of Medicine, Shahid Beheshti University of Medical Sciences, Tehran, Iran; 5 Department of Immunology, Faculty of Medicine, Tehran, Iran; 6 Department of Molecular and Cellular Biology, University of Guelph, Canada; 7 Department of Transgenic Animal Science, Stem Cell Technology Research Center, Tehran, Iran

**Keywords:** Somatic Cell Nuclear Transfer, Trichostatin A, Epigenetics Modification

## Abstract

**Objective:**

Nuclear transfer-embryonic stem cells (NT-ESCs) are genetically identical to the donor’s cells; provide a renewable source of tissue for replacement, and therefore, decrease the risk of immune rejection. Trichostatin A (TSA) as a histone deacetylase in- hibitor (HDACi) plays an important role in the reorganization of the genome and epigenetic changes. In this study, we examined whether TSA treatment after somatic cell nuclear transfer (SCNT) can improve the developmental rate of embryos and establishment rate of NT-ESCs line, as well as whether TSA treatment can improve histone modification in NT-ESCs lines.

**Materials and Methods:**

In this experimental study, mature oocytes were recovered from BDF1 [C57BL/6×DBA/2) F 1 mice] mice and enucleated by micromanipulator. Cumulus cells were injected into enucleated oocytes as donor. Reconstructed embryos were ac- tivated in the presence or absence of TSA and cultured for 5 days. Blastocysts were transferred on inactive mouse embryonic fibroblasts (MEF), so ESCs lines were estab- lished. ESCs markers were evaluated by reverse transcription-polymerase chain reaction (RT-PCR). Histone modifications were analyzed by enzyme linked immunosorbent assay (ELISA).

**Results:**

Result of this study showed that TSA treatment after SCNT can improve devel- opmental rate of embryos (21.12 ± 3.56 vs. 8.08 ± 7.92), as well as establishment rate of NT-ESCs line (25 vs. 12.5). We established 6 NT-ESCs in two experimental groups, and three embryonic stem cells (ESCs) lines as control group. TSA treatment has no effect in H3K4 acetylation and H3K9 tri-methylation in ESCs.

**Conclusion:**

TSA plays a key role in the developmental rate of embryos, establishment rate of ESC lines after SCNT, and regulation of histone modification in NT-ESCs, in a man- ner similar to that of ESCs established from normal blastocysts.

## Introduction

The pluripotent nature of embryonic stem cells (ESCs) renders them the ability to differentiate into any cell type with therapeutic potential and to hold enormous promise as tools for understanding normal development and disease, and most importantly, for cell therapy applications (1). Nuclear transfer-embryonic stem cells (NT-ESCs) are genetically identical to the donor’s cells; therefore, decrease the risk of immune rejection (2-4). Indeed, ES cells provide a renewable source of tissue for replacement, thus allow to repeat therapy when it is necessary (5). In normal development, at the time of fertilization, the oocyte and sperm nuclei are transcriptionally silent; their chromatin then undergoes extensive remodeling, accompanied by the activation of the basic transcription machinery, and leads to activate the embryonic genome (6). The molecular composition of donor nuclei In somatic cells nuclear transfer (SCNT) is different from that of egg and sperm nuclei, and their chromatin are not transcriptionally silent before transfer (7). SCNT reprograms the somatic cell genome into a totipotent cell state, and certain genomic modifications appear to undergo efficient reprogramming (8). Taken together, the available data suggest that reprogrammed cells indeed likely pose a greater risk for aggregation of harmful genomic mutations (1,9), and genes dysregulation (10,11); and this can result in the abnormalities frequently observed in cloned animals (5). It is still not completely explicit what parts of these abnormalities is due to incomplete epigenetic reprogramming or due to permanent genetic changes occur during somatic cell development or during the reprogramming process (1,12,13).

The molecular mechanisms and factors which are responsible for reprogramming and epigenetic modification are largely unknown. DNA methylation and histone modifications play serious roles in the regulation of gene activity via alterations of chromatin structure (14-16).

Evidence from various studies has indicated that chromatin is generally less compact and more ‘transcription-permissive’ in ES cells as compared with differentiated cells (17). In general, acetylation of histone H3 and H4 correlates with gene activation, while deacetylation leads to gene silencing (18). Also, methylation of H3K4 correlates with activation of chromatin, which contrasts with the modulation of inactive chromatin by methylation of H3K9 (14). Consistent with mentioned findings, chromatin in ES cell has shown high levels of acetylated H3 and H4 and di-and tri-methylated H3K4 (17).

Trichostatin A (TSA) is a histone deacetylase inhibitor (HDACi) and plays a critical role in reorganization of the chromatin and epigenetic changes in genome (19). Treatment with TSA after SCNT helps to solve the problem of genome reprogramming in cloned embryos, improves developmental rate of embryos, and also improves the rate of NT-ESCs establishment (20). But, there have been no reports about the effect of TSA treatment after SCNT on histone acetylation and methylation on NT-ESCs, yet.

In this study, we evaluated the effect of TSA on developmental rate of embryos, establishment rate of NT-ESCs lines, as well as reprogramming of two markers, acetylation of H3K9 and tri-methylation of H3K4.

## Materials and Methods

### Production of nuclear transfer oocytes and embryos

This experimental study included recipient oocytes recovered from 8- to 10-week-old B6D2F1 female mice (mating female C57 and male DBA) following superovulation with 10 IU of pregnant mare’s serum gonadotropin (PMSG) and 10 IU of human chorionic gonadotropin (hCG), 48 hours apart. Females were sacrificed 13 to 14 hours after injection of hCG and cumulus-oocyte-complexes were placed into FHM medium (MediCult, Denmark) with 300 U/ml hyaluronidase (MediCult, Denmark). After 5 minutes incubation, cumulus-denuded oocytes were collected and then washed in FHM. Groups of oocytes were enucleated in FHM medium with 5 ug/ml of cytochalasin B (Sigma,St Louis, MO, USA) washed in plain FHM, and maintained in KSOM (MediCult, Denmark) at 37˚C temperature before NT. Cumulus cells which were separated by hyaluronidase treatment were collected into 1 ml of FHM. Immediately before NT, cumulus cells were mixed with 10% clinical grade polyvinylpyrrolinde solution (MediCult, Denmark). All micromanipulations were done at room temperature using a PMM- 150 FU piezo-actuated micromanipulator unit (PrimeTech, Japan) and micro-needles shaped from borosilicate glass capillaries (21).

### Embryo culture

Following NT and a short 10 minute recovery period, oocytes were transferred to pre-activation dish containing KSOM medium with 100 nM (group 1) or without (group 2) TSA (Sigma, St Louis, MO, USA) and placed in a humidified incubator at 37˚C in 5% CO_2_ for 2 hours. After 2 hours, the oocytes were activated in 10 mM SrCl2 (Sigma, St Louis, MO, USA) and 5 ug/ml cytochalasin B-supplemented calciumfree KSOM with 100 nM or without TSA for 6 hours. After 6 hours activation, oocytes were cultured to the blastocyst stage (96 hours) into KSOM medium and placed in a humidified incubator at 37˚C in 5% CO_2_.

To provide a control group for the experiment, we mated C57 female, following superovulation with 10 IU of PMSG and 10 IU of hCG, 48 hours apart, with DBA male mice. After 3.5 days from checking vaginal plaque (VP), mice were sacrificed, and blastocysts were gain from their uterine horn (control).

### Establishment of ES cell lines

The zona pellucida (ZP) of blastocysts was removed by acid Tyrodic Acid (pH=2.5). Then, ZP free blastocysts were placed on inactive mouse embryonic ﬁbroblasts (MEF) cells.

Embryonic stem cell culture medium (ESCM) consisted of knock-Out Dulbecco’s modiﬁed Eagle’s medium (KO-DMEM) (Gibco, Germany) supplemented with nonessential amino acids (Gibco- BRL, Germany) (1%), penicillin/streptomycin (1%), L-Glutamine (2 mM), leukemia inhibitory factor (LIF) (1000 IU/ml), 2 mercaptoethanol (2 ME) (0.1 mM) and adrenocorticotropic hormone (ACTH) (10 μM) (21). ESCM was supplemented with a ﬁnal concentration of 15% fetal bovine serum (FBS) in the first day, and was replaced by serum replacement (SR).

**Fig 1:**
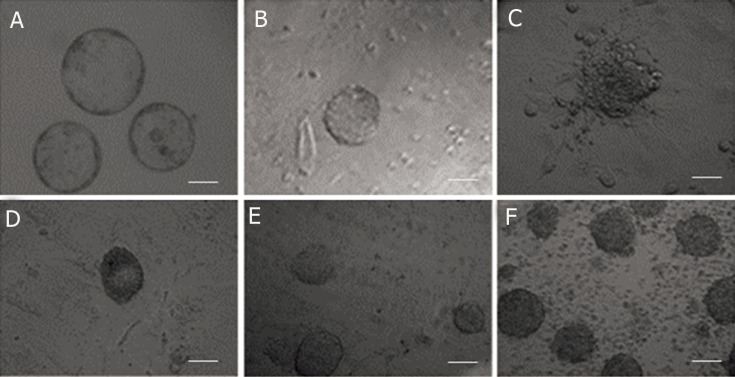
Establishment rate of ESCs line. A. SCNT blastocysts; B. Blastocyst on the MEF; C. growing ICM on MEF after 2 days; D. One colony of ESCs line (1st passage); E. ESCs line colonies (2nd passage); F. ESCs line colonies (3rd passage) (scale bar=100 μ).

After 6 days in culture, inner cell masses (ICMs) were picked and dissociated into small clumps. Then, the dissociated cells were transferred onto mouse embryonic fibroblasts (MEF). ES cell-like colonies appeared by 4-7 days of the culture (Fig 1). The colonies picked and dissociated again as described above. These cells were transferred onto new feeder cells in 25 ml flasks. Once established and expanded, cultures were passaged and frozen. All 6 NT-ESCs lines and 3 ESCs lines were passaged 7 times, while the experiments were performed on the last passage.

### Evaluation of ESCs

#### Karyotyping

For chromosome analysis, ESCs were cultured in feeder-free plates for 4-6 days. After growing to the appropriate size, ESCs colonies were treated with colcemid, hypotonic solution, and fixative, and then the slides were prepared as described in a study by PB Campos (22).

#### ESCs markers

**Fig 2:**
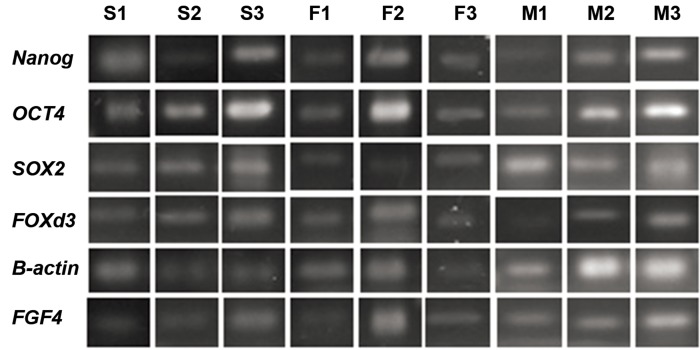
The expression profile of ESCs markers among group 1 (NT-ESCs; S1, S2, S3), group 2 (NT-ESCs; F1, F2, F3), and control (ESC; M1, M2, M3). The expression of ESCs markers were determined by RT-PCR.

We examined expression of several ESCs markers, including ß-actin, Fgf4, Foxd3, Nanog, Oct4 and Sox2, in all established ESCs lines via reverse transcription-polymerase chain reaction (RT-PCR) (Table 1,Fig 2). Besides, alkaline phosphatase activity test was performed for all ESCs based on the manufacturer’s manual of the alkaline phosphatase Kit (Sigma-Aldrich, USA).

**Table 1:** Specific primers used for PCR amplification


Genes	Forward	Reverse

**ß-actin**	CTT CTT GGG TAT GGA ATC CTG	GTG TTG GCA TAG AGG TCT TTA C
**Fgf4**	TTC GGT GTG CCT TTC TTT AC	CCG CCC GTT CTT ACT GAG
**Foxd3**	AAT CCT GGA CTC TGC TAC C	TTT ACC TGT ACG GAA AGT TAT TC
**Nanog**	TGA TTT GGT TGG TGT CTT G	TGT GAT GGC GAG GGA AG
**Oct4**	GTT CTC TTT GGA AAG GTG TTC	GCA TAT CTC CTG AAG GTT CTC


#### Histone extraction

Histone extraction was performed for histone H3 acetylation (H3K9) and H3 methylation (H3K4) based on guidelines manual of enzyme linked immunosorbent assay (ELISA) kits (Active Motif, USA).

After the colonies reached to the appropriate size, the cells were scraped, about 2×10^6^ cells were transferred to the 50 ml conical tube, and finally they were centrifuged. The cell pellets were resuspended in Lysis buffer (0.4 M HCl) and incubated on ice. After 30 minutes incubation, the cells were centrifuged, and the supernatant fraction containing acid soluble proteins was collected. The acid extracted proteins were immediately neutralized by adding phenylmethylsulfonyl fluoride (PMSF) plus dithiothreitol (DDT) as neutralization buffer. The protein concentration of acid extraction was quantified using brad ford assay and gel electrophoresis.

#### Evaluation of H3K9 acetylation and H3K4 tri-methylation

Evaluation of histone H3 acetylation and histone H3 methylation was performed as guidelines manual for Histone H3 acetyl Lys9 (Active Motif, catalog No.53114) and the Histone H3 methylated Lys4 of ELISA kit (Active Motif, catalog No. 53113); briefly, the standard protein and samples were added to the capture plate, followed by 1 hour incubation at room temperature. Then, the first diluted antibody was added to the capture plate as in guidelines manual. After incubating and washing with buffer, the second diluted antibody was added, incubated at room temperature for 1 hour, and washed with buffer. Finally, developing solution was added in order to record absorbance values using spectrophotometer.

#### Statistical analysis

Comparison between developmental rates of embryos and establishment rate of ESCs lines was performed by non-parametric analysis. Comparison of histone acetylation and tri-methylation between ESCs groups was performed by ANOVA. All statistical analysis was performed using SPSS

#### Ethical considerations

All experiments and protocols were strongly performed according to the Guiding Principles for the Care and Use of Research Animals adopted by the Shahid Beheshti University of Medical Science and Stem Cell Research Center Committee on Animal Research and Bioethics.

## Results

### Production of cloned and fertilized embryos and establishment of ESC lines

**Fig 3:**
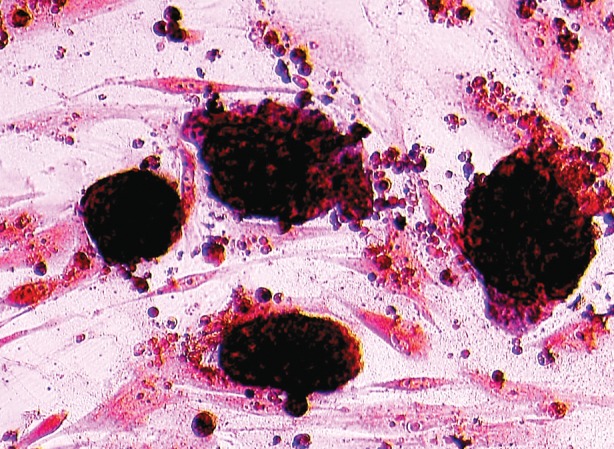
Alkaline phosphatase test; ESCs colonies with positive alkaline phosphatase test became brown or red.

The developmental rates of embryo derived from SCNT are shown in table 2. Comparison of two groups showed that there is a significant difference between developmental rates after SCNT with or without TSA treatment. We established 3 embryonic stem cell lines from SCNT blastocysts in each group (Fig 1a-c). The karyotypes of all established ESC lines were up to 80% normal (23). ESCs markers (Nanog, OCT4, SOX2, FOXd3, ßactin, and FGF4) were expressed in all ESC lines (Fig 2), and the results of the alkaline phosphatase test were positive for every group (Fig 3).

**Table 2:** Developmental rate of embryos and establishment rate of ESC lines in group 1, 2, and control


Groups	2 PN	2 cells	4 cells	8 cells	Morulla	Blastocyst	ESC line

**Control**	-	-	-	-	-	11	3(27.3)^s^
**Group 1**	55	44(81.86 ± 13.76)^a^	34(63.97 ± 17.41)^b^	26(50.38 ± 20.19)^c^	20(37.97 ± 17.23)^d^	12(21.12 ± 3.56)^e^	3(25)^f^
**Group 2**	258	162(62.32 ± 9.27)^a^	90(34.09 ± 7.31)^b^	66(24.9 ± 6.16)	36(12.12 ± 12.51)^d^	24(8.08 ± 7.92)^e^	3 (12.5)^f^^s^


Identical alphabetic letters show the significant differences among groups (p<0.05).

### Evaluation of H3K9 acetylation and H3K4 methylation

**Fig 4:**
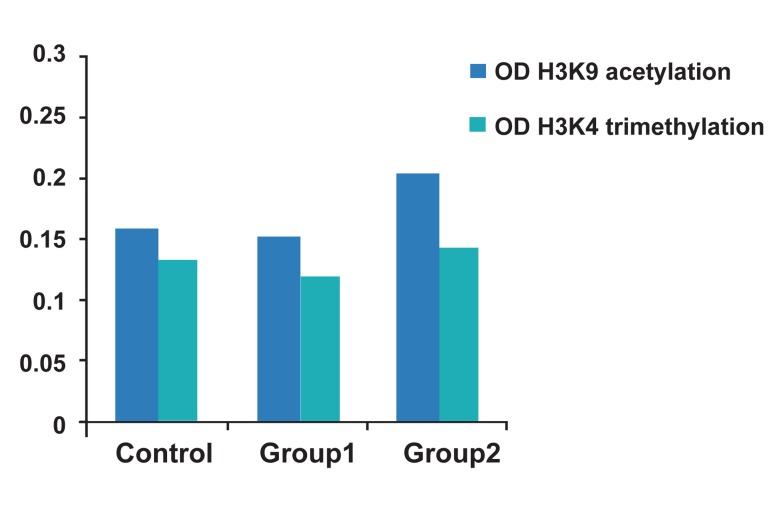
Comparison of H3 acetylation (H3K9) and H3 methylation (H3K4) among three groups.

A total of 9 ESC lines, 2 experimental groups and one control group, were examined in this study. The different absorbance values taken by spectrophotometer for histone H3 acetylation (H3K9) and H3 methylation (H3K4) in group 1, 2, and control were shown in figure 4. There are no significant differences between experimental and control groups for histone H3 acetylation and methylation.

## Discussion

NT-ESCs are genetically identical to the donor of the nucleus, thus are useful for therapeutic applications (4). In this study, we examined whether TSA treatment after SCNT can improve developmental rate of embryos and establishment rate of NTESCs line, as well as whether TSA treatment can improve histone modification in NT-ESCs lines. The developmental rate of blastocysts and the establishment rate of ESC line were significantly higher in the group which was treated by TSA after SCNT. Several studies have shown the same results about the developmental rate of blastocyst using treatment with TSA after SCNT (20,24-29). This result suggests that nuclear reprogramming might be rise by chemical treatment, and that TSA treatment improves the SCNT technique and provides new insights into the genomic reprogramming of somatic cell nuclei (30). Positive effect of TSA treatment can be the result of improved regulation of epigenetic processes associated with reprogramming of somatic genome (20,31). TSA, as an HDACi, induces histone hyperacetylation (32,33). This may consequently enhance the regulation of DNA methylation (20,34,35), while improving the reprogramming (28), so gene expression in 2-cell stage SCNT embryos after TSA treatment is higher than non-treated 2-cell stage SCNT embryos, and is the same as gene expression in in vitro fertilization (IVF) embryos. This is the key reason for improved developmental rates of SCNT embryos (27).

A number of studies have shown development of lysine acetylation on core histones in TSAtreated embryos generated from SCNT in a manner similar to that in normal embryos (28,36,37). And also, the treatment of oocyte with TSA after SCNT causes an increase in the level of methylation of histone H3 at K4 in SCNT-generated embryos (29). There is not enough information about genomic reprogramming involving histone methylation in embryos. Specially, the effect of TSA treatment on histone methylation in cloned embryos is still unknown (29), with the exception of one study which has reported TSA treatment has no effect on the distribution and disappearance of H3-K9 tri-methylation in cloned embryos (38).

Consistent with the positive effect of TSA on improvement of embryo development, our findings revealed that NT-ESCs from TSA-treated clones were established two times more than from control clones. Our result is consistent with the only study that investigated the effect of TSA treatment on establishment of ESC line after SCNT (20). We showed that NT-ESCs are equivalent, in normal karyotype and ESCs marker expression, to the normal ESCs, while it is consistent with the other studies (20,39,40). As we discussed about the positive effect of TSA on developmental rate of embryos, gene expression, and also histone modification after SCNT, TSA might enhance the establishment rate of NT-ESCs line.

In the present study, for the first time, we showed improved histone modification in TSA-treated ESCs lines, so that the pattern of histone acetylation and methylation is similar to ESCs lines established from normal blastocysts. These finding is correlated with positive effect of TSA on histone modification of cloned embryos. Although histone acetylation and tri-methylation in group 2 was higher than the group1 and control group, there were no significant differences between experimental and control groups.

The chromatin of ESCs is generally more transcription- permissive and less compact in comparison with differentiated cells (17). For instance, differentiation of human and mouse ES cells leads to increase deacetylation of histone H4 (41). In general, in all types of cells, including differentiated and non-differentiated cells, acetylation of histone H3 and H4 correlates with gene activation, while deacetylation leads to gene silencing (18). Moreover, methylation of H3K4 is related to activation of chromatin, which contrasts with the modulation of inactive chromatin by methylation of H3K9 (14). Consistent with mentioned findings, ESCs chromatin have shown high levels of acetylated H3 and H4 and di-and tri-methylated H3K4 (17). And also, ESCs show a markedly increased exchange rate, in H2B and H3 and the heterochromatin-associated protein HP1, which binds di- and tri-methylated histone H3 at lysine 9 (H3K9), compared with differentiated cells (42). But, there are not any reports about the effect of TSA on histone modification in NT-ESCs.

The most important role which TSA play in SCNT is improvement of genome reprogramming. TSA promotes genome reprogramming in embryos through histone methylation after SCNT. Just as normally fertilized embryos showing high levels of dime-H3-K4, in the same manner, TSA treatment causes an increase in the levels of dime-H3- K4 in SCNT-generated embryos (29) and histone methylation at K4 correlated with gene promoter activity (43,44). Therefore, the increased levels of dime-H3-K4 in control and TSA-treated SCNT embryos can be a reason for enhancing chromosome decondensation and transcription activity of the embryos, which leads to a more accurate regulation of embryo development (29,45). In the present study, although there were no significant difference in histone modification between TSAtreated and non-treated ESCs lines, the pattern of histone acetylation and methylation in non-treated ESCs lines was different from the TSA-treated group showing higher level of H3K4 tri-methylation and H3K9 acetylation. As TSA can improve reprogramming after SCNT, deficient reprogramming in the non-treated group can be the reason for the different histone tri-methylation and acetylation pattern from TSA-treated and control groups. As in this experiment, by treating the oocytes after SCNT, we could establish the NT-ESCs lines following the histone modification pattern in the same manner of ESCs lines established from normal blastocysts.

## Conclusion

We can say that TSA plays an important role in the developmental rate of embryos and establishment of ESC lines after SCNT, as well as in the regulation of histone modification in NT-ESCs, in the similar manner of ESCs established from normal blastocysts.

## References

[1] Puri MC, Nagy A (2012). Concise review: embryonic stem cells versus induced pluripotent stem cells: the game is on. Stem Cells.

[2] Solter D, Gearhart J (1999). Putting stem cells to work. Science.

[3] Lanza RP, Cibelli JB, West MD (1999). Human therapeutic cloning. Nat Med.

[4] Colman A, Kind A (2000). Therapeutic cloning: concepts and practicalities. Trends Biotechnol.

[5] Hochedlinger K, Rideout WM, Kyba M, Daley GQ, Blelloch R, Jaenisch R (2004). Nuclear transplantation, embryonic stem cells and the potential for cell therapy. Hematol J.

[6] Latham KE (1999). Mechanisms and control of embryonic genome activation in mammalian embryos. Int Rev Cytol.

[7] Solter D (2000). Mammalian cloning: advances and limitations. Nat Rev Genet.

[8] Eggan K, Akutsu H, Hochedlinger K, Rideout W, Yanagimachi R, Jaenisch R (2000). X-Chromosome inactivation in cloned mouse embryos. Science.

[9] Ramos-Mejia V, Munoz-Lopez M, Garcia-Perez JL, Menendez P (2010). iPSC lines that do not silence the expression of the ectopic reprogramming factors may display enhanced propensity to genomic instability. Cell Res.

[10] Humpherys D, Eggan K, Akutsu H, Hochedlinger K, Rideout WM, Biniszkiewicz D (2001). Epigenetic instability in ES cells and cloned mice. Science.

[11] Humpherys D, Eggan K, Akutsu H, Friedman A, Hochedlinger K, Yanagimachi R (2002). Abnormal gene expression in cloned mice derived from embryonic stem cell and cumulus cell nuclei. Proc Natl Acad Sci USA.

[12] Mayshar Y, Ben-David U, Lavon N, Biancotti JC, Yakir B, Clark AT (2010). Identification and classification of chromosomal aberrations in human induced pluripotent stem cells. Cell Stem Cell.

[13] Hussein SM, Batada NN, Vuoristo S, Ching RW, Autio R, Narva E (2011). Copy number variation and selection during reprogramming to pluripotency. Nature.

[14] Lachner M, O’sullivan RJ, Jenuwein T (2003). An epigenetic road map for histone lysine methylation. J Cell Sci.

[15] Mikkelsen TS, Ku M, Jaffe DB, Issac B, Lieberman E, Giannoukos G (2007). Genome-wide maps of chromatin state in pluripotent and lineage-committed cells. Nature.

[16] Hezroni H, Tzchori I, Davidi A, Mattout A, Biran A, Nissim- Rafinia M (2011). H3K9 histone acetylation predicts pluripotency and reprogramming capacity of ES cells. Nucleus.

[17] Spivakov M, Fisher AG (2007). Epigenetic signatures of stem-cell identity. Nat Rev Genet.

[18] Fry CJ, Peterson CL (2001). Chromatin remodeling enzymes: who’s on first?. Curr Biol.

[19] Meshorer E, Misteli T (2006). Chromatin in pluripotent embryonic stem cells and differentiation. Nat Rev Mol Cell Biol.

[20] Kishigami S, Mizutani E, Ohta H, Hikichi T, Thuan NV, Wakayama S (2006). Significant improvement of mouse cloning technique by treatment with trichostatin A after somatic nuclear transfer. Biochem Biophys Res Commun.

[21] Ogawa K, Matsui H, Ohtsuka S, Niwa H (2004). A novel mechanism for regulating clonal propagation of mouse ES cells. Genes Cells.

[22] Campos PB, Sartore RC, Abdalla SN, Rehen SK (2009). Chromosomal spread preparation of human embryonic stem cells for karyotyping. J Vis Exp.

[23] Men H, Bauer BA, Bryda EC (2012). Germline transmission of a novel rat embryonic stem cell line derived from transgenic rats. Stem Cells Dev.

[24] Thuan NV, Kishigami S, Wakayama T (2010). How to improve the success rate of mouse cloning technology. J Reprod Dev.

[25] Kishigami S, Bui HT, Wakayama S, Tokunaga K, Van Thuan N, Hikichi T (2007). Successful mouse cloning of an outbred strain by trichostatin A treatment after somatic nuclear transfer. J Reprod Dev.

[26] Rybouchkin A, Kato Y, Tsunoda Y (2006). Role of histone acetylation in reprogramming of somatic nuclei following nuclear transfer. Biol Reprod.

[27] Shao GB, Ding HM, Gao WL, Li SH, Wu CF, Xu YX (2009). Effect of trychostatin A treatment on gene expression in cloned mouse embryos. Theriogenology.

[28] Li J, Svarcova O, Villemoes K, Kragh PM, Schmidt M, Bogh IB (2008). High in vitro development after somatic cell nuclear transfer and trichostatin A treatment of reconstructed porcine embryos. Theriogenology.

[29] Bui HT, Wakayama S, Kishigami S, Park KK, Kim JH, Thuan NV (2010). Effect of trichostatin A on chromatin remodeling, histone modifications, DNA replication, and transcriptional activity in cloned mouse embryos. Biol Reprod.

[30] Wakayama S, Wakayama T (2010). Improvement of mouse cloning using nuclear transfer-derived embryonic stem cells and/or histone deacetylase inhibitor. Int J Dev Biol.

[31] de Ruijter AJ, van Gennip AH, Caron HN, Kemp S, van Kuilenburg AB (2003). Histone deacetylases (HDACs): characterization of the classical HDAC family. Biochem J.

[32] Nervi C, Borello U, Fazi F, Buffa V, Pelicci PG, Cossu G (2001). Inhibition of histone deacetylase activity by trichostatin A modulates gene expression during mouse embryogenesis without apparent toxicity. Cancer Res.

[33] Yoshida M, Kijima M, Akita M, Beppu T (1990). Potent and specific inhibition of mammalian histone deacetylase both in vivo and in vitro by trichostatin A. J Biol Chem.

[34] Cervoni N, Szyf M (2001). Demethylase activity is directed by histone acetylation. J Biol Chem.

[35] Geiman TM, Robertson KD (2002). Chromatin remodeling, histone modifications, and DNA methylation-how does it all fit together?. J Cell Biochem.

[36] Iager AE, Ragina NP, Ross PJ, Beyhan Z, Cunniff K, Rodriguez RM (2008). Trichostatin A improves histone acetylation in bovine somatic cell nuclear transfer early embryos. Cloning Stem Cells.

[37] Shi LH, Ai JS, Ouyang YC, Huang JC, Lei ZL, Wang Q (2008). Trichostatin A and nuclear reprogramming of cloned rabbit embryos. J Anim Sci.

[38] Wang F, Kou Z, Zhang Y, Gao S (2007). Dynamic reprogramming of histone acetylation and methylation in the first cell cycle of cloned mouse embryos. Biol Reprod.

[39] Wakayama S, Jakt ML, Suzuki M, Araki R, Hikichi T, Kishigami S (2006). Equivalency of nuclear transfer-derived embryonic stem cells to those derived from fertilized mouse blastocysts. Stem Cells.

[40] Wakayama T, Tabar V, Rodriguez I, Perry AC, Studer L, Mombaerts P (2001). Differentiation of embryonic stem cell lines generated from adult somatic cells by nuclear transfer. Science.

[41] Ramalho-Santos M, Yoon S, Matsuzaki Y, Mulligan RC, Melton DA (2002). "Stemness": transcriptional profiling of embryonic and adult stem cells. Science.

[42] Gilbert N, Thomson I, Boyle S, Allan J, Ramsahoye B, Bickmore WA (2007). DNA methylation affects nuclear organization, histone modifications, and linker histone binding but not chromatin compaction. J Cell Biol.

[43] Liang G, Lin JC, Wei V, Yoo C, Cheng JC, Nguyen CT (2004). Distinct localization of histone H3 acetylation and H3-K4 methylation to the transcription start sites in the human genome. Proc Natl Acad Sci USA.

[44] Schneider R, Bannister AJ, Myers FA, Thorne AW, Crane- Robinson C, Kouzarides T (2004). Histone H3 lysine 4 methylation patterns in higher eukaryotic genes. Nat Cell Biol.

[45] Kretsovali A, Hadjimichael C, Charmpilas N (2012). Histone deacetylase inhibitors in cell pluripotency, differentiation, and reprogramming. Stem Cells Int.

